# The effect of geo-climatic determinants on the distribution of cutaneous leishmaniasis in a recently emerging focus in eastern Iran

**DOI:** 10.1186/s13071-021-05046-0

**Published:** 2021-10-15

**Authors:** Mehdi Karamian, Mohammad Amin Ghatee, Majid Shayesteh, Walter Robert Taylor, Saeed Mohebi-Nejad, Ghasem Taheri, Mohammad Reza Jamavar

**Affiliations:** 1 Department of Parasitology and Mycology, School of Medicine, Zanjan University of Medical Sciences, Zanjan, Iran; 2 Cellular and Molecular Research Center, Yasuj University of Medical Sciences, Yasuj, Iran; 3 Department of Parasitology, School of Medicine, Yasuj University of Medical Sciences, Yasuj, Iran; 4 Center for Disease Control, Birjand University of Medical Sciences, Birjand, Iran; 5 Mahidol Oxford Tropical Medicine Research Unit, Bangkok, Thailand; 6 Oxford Centre for Tropical Medicine and Global Health, University of Oxford, Oxford, UK; 7 Student Research Committee, Birjand University of Medical Sciences, Birjand, Iran

**Keywords:** Cutaneous leishmaniasis, Emerging focus, GIS, Geo-climatic determinants

## Abstract

**Background:**

Cutaneous leishmaniasis (CL) has been reported in recent years in South Khorasan Province, a desert region of eastern Iran, where the main species is *Leishmania tropica*. Little is known of the influence of geography and climate on its distribution, and so this study was conducted to determine geo-climatic factors by using geographic information system.

**Methods:**

The home addresses of patients with CL patients who were diagnosed and notified from 2009 to 2017 were retrieved from the provincial health center and registered on the village/town/city point layer. The effects of mean annual rainfall (MAR) and mean annual humidity (MAH), mean annual temperature (MAT), maximum annual temperature (MaxMAT), minimum annual temperature (MinMAT), mean annual number of high-velocity wind days (MAWD), mean annual frosty days (MAFD) and snowy days (MASD), elevation, soil type and land cover on CL distribution were examined. The geographical analysis was done using ArcMap software, and univariate and multivariate binary logistic regression were applied to determine the factors associated with CL.

**Results:**

A total of 332 CL patients were identified: 197 (59.3%) male and 135 (40.7%) female. Their mean age was 29.3 ± 2.1 years, with age ranging from 10 months to 98 years. CL patients came from a total of 86 villages/towns/cities. By multivariate analysis, the independent factors associated with increased CL were urban setting (OR = 52.102), agricultural land cover (OR = 3.048), and MAWD (OR = 1.004). Elevation was a protective factor only in the univariate analysis (OR = 0.999). Soil type, MAH, MAT, MinMAT, MaxMAT, and MAFD did not influence CL distribution in eastern Iran.

**Conclusions:**

The major risk zones for CL in eastern Iran were urban and agricultural areas with a higher number of windy days at lower altitudes. Control strategies to reduce human vector contact should be focused in these settings.

**Graphical abstract:**

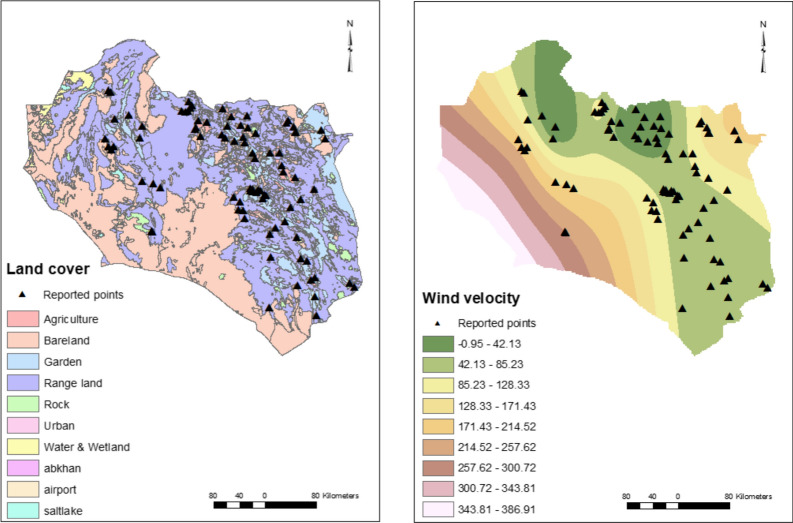

## Background

Leishmaniasis is a disease complex caused by various species of *Leishmania*, a parasite that is transmitted by sand flies. Clinically, leishmaniasis may manifest across a spectrum of symptoms, from self-limiting cutaneous disease (CL) to fatal visceral (VL) forms [1]. Individuals in more than 100 countries and territories are at risk of leishmaniasis, and about 1.5 million new patients are reported annually [2]. CL is the most prevalent form, and the majority of CL cases, accounting for 84% of the global burden, occur in the Middle East (Afghanistan, Iran, Iraq, Syria, Pakistan, and Yemen), North Africa (Tunisia and Algeria), and South America (Brazil, Peru, and Colombia). *Leishmania tropica* and *L. major* are the main etiological species of Old World CL, but *L. aethiopica*, *L. infantum*, and *L. donovani* have also been isolated from CL lesions in some countries [3]. *Leishmania major* has a wide geographical distribution from Central Asia to the Middle East, the Mediterranean Basin, and parts of West Africa, whilst *L. tropica* is found mostly in the Middle East and the Eastern Mediterranean, with foci in Africa and some regions of southern and central Asia [1].

The epidemiology of leishmaniasis depends on interactions between the parasite, vector, host, and the environment [4]. In Iran, CL is caused by *L. major* and *L. tropica*, with an estimated annual incidence of 20,000 cases [5]. *Leishmania major* causes zoonotic CL (ZCL), which is transmitted mostly by *Phlebotomus papatasi* sand flies to humans from desert rodents of the Gerbillidae family, including *Rhombomys opimus*, *Tatera indica*, *Meriones libycus*, *M. persicus*, *M. hurrianae*, and *Nesokia indica* in Iran [6]. *Leishmania tropica*, the main etiological species of anthroponotic CL, is transmitted between humans by *Ph. sergenti*, but it has also been isolated rarely from dogs in Iran [7]. Although *L. major* and *L. tropica* overlap in most endemic foci of Iran, *L. major* is dominant in the southwestern, western, central, and northeastern regions, whilst most cases of *L. tropica* have been reported from large cities and some small towns and villages in the southeast (Kerman Province) and east (South Khorasan Province), as well as in certain large cities in the southwest (Shiraz), northeast (Mashhad), and central regions (Tehran) [3, 8].

Geospatial information systems (GIS) and remote sensing (RS) are useful tools for mapping and modeling of diseases and determining key geo-climatic factors and man-made environmental changes [9]. In recent years, GIS research has established a close link between increasing CL distribution and climatic and environmental factors in southwestern [10], northern [11], and central [12] Iran. South Khorasan Province is an emerging focus of CL in eastern Iran, where, in common with the bordering western areas of Afghanistan, it is caused mainly by *L. tropica* [13]. Most of the province is desert, and to date, no GIS study has examined climatic and environmental factors associated with CL burden. We, therefore, conducted such a study and report the findings herein.

## Methods

### Study area

South Khorasan in eastern Iran is the third largest province of Iran and comprises 11 counties, with a surface area of 151,913 km^2^. It lies between 57.43–61.04 E longitude and 34.04–30.15 N latitude in eastern Iran. It has a predominantly desert climate with a range of annual precipitation from 86 to 186 mm and a wide temperature range from −13.5 to 46.8 °C [14]. The main agricultural products of South Khorasan include wheat (*Triticum*), followed by barley (*Hordeum vulgare*) and sugar beet (*Beta vulgaris*). Saffron (*Crocus sativus*), barberries (*Berberis vulgaris*), wild olive (*Elaeagnus angustifolia*), and jujube (*Ziziphus jujube*) are also produced. There are more than 5000 residential properties in the province, and the capital city is Birjand. CL has been emerging in recent decades, and *L*. *tropica* is the predominant species [13].

### Patient data

CL is a notifiable disease in Iran. Reports are submitted by the public and private sectors as clinically suspected or confirmed cases depending on the diagnostic facilities available (e.g. Giemsa/Leishman-stained skin scraping and polymerase chain reaction [PCR]). Reports are sent to health centers located in different parts of the province and then collected at the provincial health center. We obtained demographic data and the home addresses of all properly recorded cases reported between 2009 and 2017 in South Khorasan Province.

### Geospatial data

Patient addresses were mapped on a point shapefile of villages, towns, and cities of South Khorasan Province. Meteorological data including temperature, humidity, evaporation, the number of days with high-velocity wind, frosty days, and snowy days from 10 synoptic stations (Birjand, Ghaen, Tabas, Ferdos, Nehbandan, Sarayan, Haji Abad, Boshrouyeh, Khour, and Sarbisheh) were retrieved from the South Khorasan Provincial Weather Bureau. Rainfall data were obtained from all 38 rainfall stations from across the province. The mean annual temperature (MAT), mean maximum annual temperature (MaxMAT), mean minimum annual temperature (MinMAT), mean annual rainfall (MAR), mean annual humidity (MAH), mean annual high-velocity wind days (MAWD), mean annual frosty days (MAFD), and mean annual snowy days (MASD) were calculated for the study period.

The precision of different interpolation methods was checked for generating the meteorological raster layers. The MAR and MAWD were generated by kriging and tension-based spline interpolation, respectively. The remaining meteorological layers were developed using the inverse distance weighted (IDW) interpolation method, and a grid with resolution of 1 × 1 km was used for the interpolations. The raster layer of the digital elevation model (DEM) and the vector layer of land cover, including surface cover features, were obtained from the Department of Agricultural Affairs (https://kj-agrijahad.ir).

### Geo-climatic analysis

Villages/towns/cities of South Khorasan Province as the basic point layer were extracted with the raster layers of MAT, MinMAT, MaxMAT, MAR, MAH, MASD, MAFD, MAWD, and DEM. The geometric intersection of the layer developed from the extraction of the abovementioned layers with the land cover polygonal vector file was computed by the identity tool to generate the final layer in which each point showed properties of all the overlapped features from the raster and vector layers. The attribute of this layer was converted to an Excel format (Microsoft 2013) for statistical analysis. All geo-climatic analyses were accomplished using ArcGIS version 10.5 (http://www.esri.com/arcgis).

### Statistical analysis

Statistical analysis was based on the presence or absence of CL patients in each village/town/city point. The effects of the climatic and environmental factors on CL distribution in the study areas were assessed by univariate and multivariate logistic regression models, using SPSS version 21 software.

## Results

A total of the 332 CL patients were identified; 59.3% (*n* = 197) were male and 40.7% (*n* = 135) female. Their mean age was 29.3% ± 2.1 years, with age ranging from 10 months to 98 years. CL patients came from a total of 86 villages/towns/cities. Although the highest number of CL patients was reported from Birjand (*n* = 94), the highest incidence (*N*/1000) was reported in Masoudi village (71.42), followed by Mohammadabad (52.63), Zardgah (51.94), Sourg (30.92), Golnam-olia (17.54), Esfak (17.24), and Karijgan (16.94). The lowest incidence rates were reported from Nehbandan city and Tabas-Mesina village (0.21).

### Univariate analysis

#### Climatic factors

**Fig. 1:**
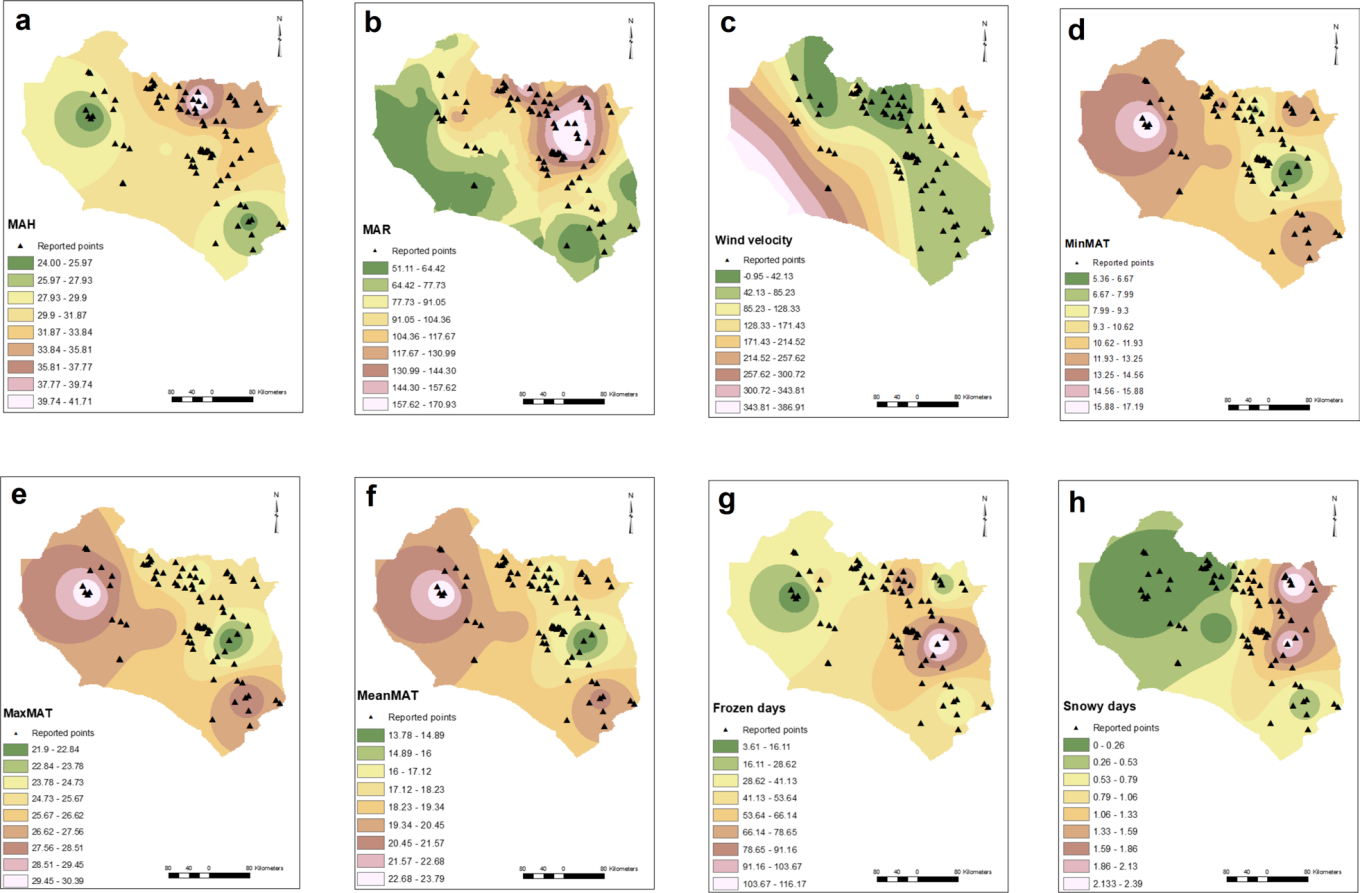
The climatic interpolated raster maps: mean annual humidity (**a**), mean annual rainfall (**b**), mean annual high-velocity wind days (**c**), mean annual minimum temperature (**d**), mean maximum annual temperature (**e**), mean annual temperature (**f**), mean annual frosty days (**g**), and mean annual snowy days (**h**). The number of days with high wind velocity showed a weak effect on CL occurrence whilst other climatic factors had no effect on CL in South Khorasan Province

This analysis showed that MAWD was a factor (OR = 1.004, *P* = 0.043, 95% CI 1–1.008), increasing the probability of CL by 0.4%/day, whilst MAFD, MASD, MAT, MinMAT, MaxMAT, and MAR were not significant factors (Table 1). There was a decreasing trend for MAH (OR = 0.939, *P* = 0.072, 95% CI 0.877–1.006) (Fig. 1).

**Table 1:** Univariate analysis of the effect of climatic factors on CL in South Khorasan Province

Variable	*P*-value	OR	95% CI
MAWD	0.043	1.004	1–1.008
MAFD	0.568	0.997	0.986–1.008
MASD	0.429	1.137	0.827–1.563
MAT	0.533	1.039	0.921–1.172
MinMAT	0.665	1.022	0.925–1.130
MaxMAT	0.372	1.065	0.928–1.223
MAR	0.301	0.996	0.988–1.004
MAH	0.072	0.939	0.877–1.006

#### Environmental factors

**Fig. 2:**
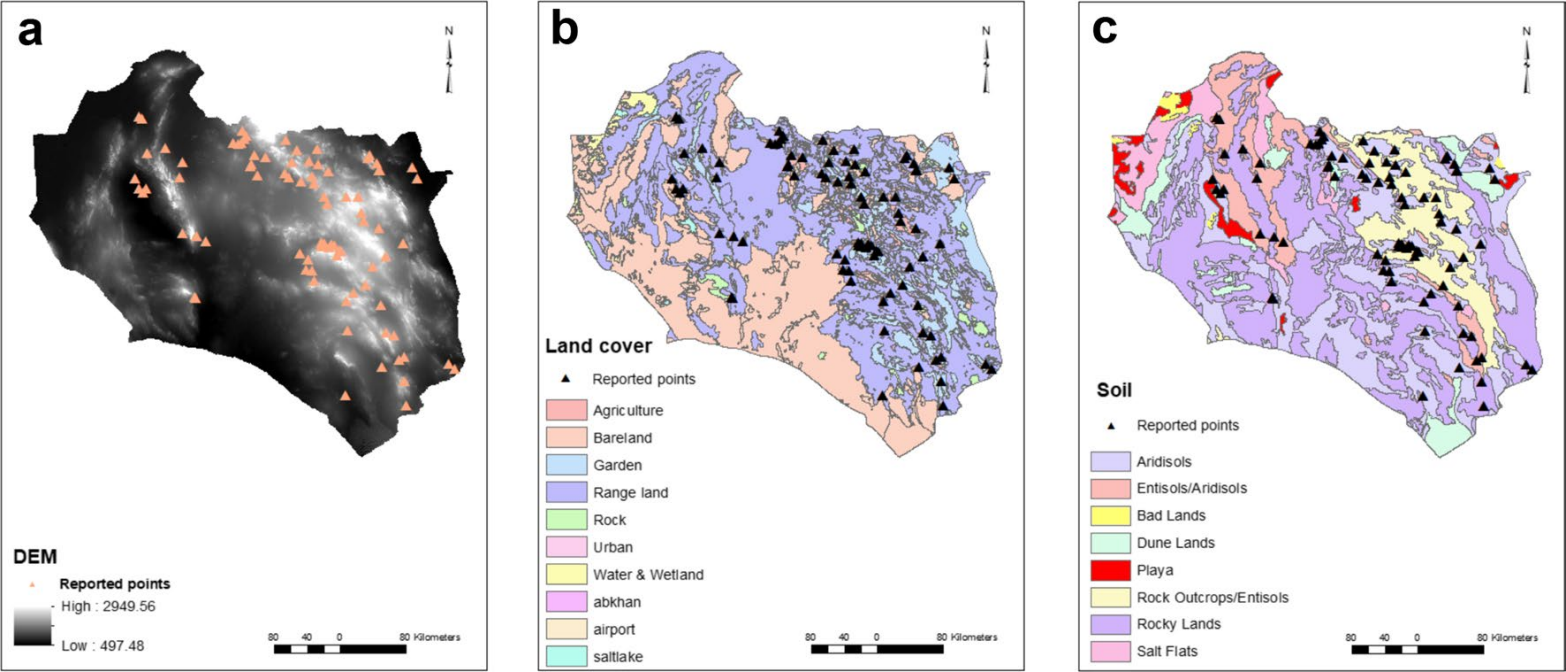
Maps of the environmental factors: digital elevation model (**a**), land cover (**b**), and soil types (**c**). Urban and agricultural land cover showed direct positive effects whilst elevation had a negative effect on CL occurrence. Soil type was not associated with CL in South Khorasan Province

Urban setting and agricultural land cover were significant explanatory variables, increasing the odds of CL 49- and 2.8-fold, respectively (Table 2). Rangeland, rock, and bare land were not significant. No soil types, including aridisols, salt flats, rocky land, rock outcrops/entisols, playa, and dune land, were associated with CL occurrence. Elevation significantly reduced CL by 1% for each meter increase in altitude (Fig. 2).

**Table 2:** Univariate analysis of the effects of environmental factors on CL in South Khorasan Province

Variable	*P*-value	OR	95% CI
Soil class			
Dune land (constant)	0.000		
Entisols/Aridisols	0.462	2.153	0.279–16.6290
Aridisols	0.252	3.207	0.437–23.555
Salt flats	0.532	2.160	0.193–24.190
Rocky land	0.677	1.539	0.203–11.676
Rock outcrops/entisols	0.941	0.926	0.12–7.141
Playa	1	0.000	
Land cover			
Garden (constant)	0.000		
Agriculture	0.005	2.769	1.358–5.646
Rangeland	0.773	0.894	0.420–1.906
Rock	0.74	1.42	0.179–11.256
Urban	0.000	49	20.888–114.947
Bare land	0.912	1.068	0.333–3.427
DEM	0.022	0.999	0.999–1

### Multivariate analysis

To reveal the concomitant effects, multivariate binary logistic regression was applied to analyze the factors shown to be significantly associated with CL occurrence by univariate analysis. By multivariate analysis, the associated factors were urban setting (OR = 52.102, *P* < 0.001, 95% CI 20.765–130.727), agricultural land cover (OR = 3.048, *P* = 0.005, 95% CI 1.411–6.585), and MAWD (OR = 1.004, *P* = 0.041, 95% CI 1–1.009); elevation lost its significance (Table 3).

**Table 3:** Multivariate analysis of geo-climatic factors associated with CL in South Khorasan Province

Variable	*P*-value	OR	95% CI
Land cover			
Garden (constant)	0.000		
Agriculture	0.005	3.048	1.411–6.585
Rangeland	0.781	0.897	0.415–1.939
Rock	0.839	1.241	0.154–10.022
Urban	0.000	52.102	20.765–130.727
Bare land	0.923	1.061	0.320–3.512
DEM	0.45	1	1–1.001
MAWD	0.041	1.004	1–1.009

## Discussion

We have shown that in South Khorasan, a mostly dry province, urban land cover was associated with the highest odds of acquiring CL, followed by agricultural land and MAWD. Elevation was a factor but only in the univariate analysis. There was no association with temperature, rainfall, frost, snow, soil type, most land features, or humidity.

Urban areas (large residential regions including cities, towns, and large villages) are determined primarily by remote sensing data. Urban settings are well known to be associated with CL and VL in Iran and have been identified as the most important factor affecting the distribution of CL in western [15] and southwestern [10] Iran. A GIS-based study of VL caused by *L. infantum* in Iran [10] also reported a significant effect of living in an urban area [16]. A systematic review in 2020 concluded that although during the first half of the twentieth century, living in areas away from population centers or in proximity to forests were important risk factors for leishmaniasis, over time, urban and peri-urban dwellers were at the greatest risk of leishmaniasis. Vector adaptation to urban areas and the expansion of these areas to surrounding vegetation, and proximity to agricultural areas as natural breeding grounds for vectors and probable reservoirs, especially in developing countries, increase the chances of acquiring leishmaniasis [17]. In addition, buildings and construction sites in villages, towns, and cities offer breeding sites for urban-adapted sand flies, and increased vector populations increase the probability of CL transmission. Another important aspect of CL transmission is human population density, which is higher in urban areas, and this favors the transmission of anthroponotic CL caused by *L. tropica* [18]. Moreover, population migration from remote rural areas to urban suburbs for work and residence increases the probability of CL [19].

Agricultural land cover was another key determinant of CL distribution, and this is probably related to human activity within these areas. Although South Khorasan has a desert climate, there is much agricultural activity and market gardening. Orchards have been established for thousands of years around limited water sources, but prolonged drought in recent decades has concentrated activity in fewer areas that are close to population centers [20]. The causative agent of CL in South Khorasan has been identified as *L. tropica* in over 95% of cases [13], and this supports the notion that increased human activity has resulted in increased CL.

Other studies have reported findings similar to ours. Urban and dry farm settings have also been identified as factors for CL transmission in southwestern Iran [10]. An outbreak of CL and VL in Spain was associated with the construction of a park in a suburb of Madrid [21], whilst in Pakistan, CL was active in the lowlands of Chitral district, where urbanization and deforestation were prominent [22]. Green urban areas were also associated with the spread of VL in Greece [23]. In a systematic review, city expansion that encroached on the “green belt,” resulting in pockets of “green” within semi-urban areas, was identified as an important hazard for increasing CL spread. Close proximity of vegetation and urban setting were determining factors for the spread of CL in 63% of the studies in this systematic review [17].

We found that an increased number of windy days was associated with increased CL in eastern Iran. Almost all studies in this field have focused more on wind velocity than the number of days with high-velocity wind. However, Galgamuwa et al. [24] also showed a direct effect of wind speed on leishmaniasis in Sri Lanka, consistent with two studies from central [25] and southern [26] Iran, with Ramezankhani et al. [25] demonstrating that a wind speed of 12–16 m/s increased the spread of CL. By contrast, wind was not a risk factor for CL [27] and VL [28] in southwestern and northwestern Iran, respectively. South Khorasan contains the central and southern Iranian Kavirs (huge deserts) that are characterized by high-velocity desert winds. Wind affects sand flies and CL in several ways: sand flies may be unable to maintain their bite on human/animal skin, they may be blown to more distant areas to establish new breeding foci, and they may seek shelter in homes or animal barns, thereby increasing vector human/animal contact [29]. More research is needed to confirm these hypotheses and inform control programs.

Elevation was inversely associated with CL in this region, where the mean elevation in village/cities with reports of CL was 1399 ± 409 m, and points with no reports showed a mean altitude of 1502 m (not shown in results).

Hanafi-Bojd et al. [30] reported the presence of *Ph. sergenti* and *Ph. papatasi*, the vectors of *L. tropica* and *L. major*, at ~ 1235 m and just under 1000 m, respectively, in different regions of Iran. *Leishmania tropica* is dominant in eastern Iran, where the elevation favors *Ph. sergenti*. Similar findings have been reported from other studies in Iran [10], Afghanistan [31], Brazil [32], and Spain [33], although one study from northeastern Iran did not find an association between elevation and CL risk [34]. It seems that one reason for this relationship is that cities/towns/villages are located at lower altitudes, especially in mountainous regions [10]. Furthermore, in dry desert conditions, as found in eastern Iran, the location of human settlements is determined by the limited availability of water resources, which mostly consist of underground water such as wells and qanats in the lowlands.

We did not find an independent association with rainfall but there was a trend for humidity. Variable results have been found for the effect of rainfall on leishmaniasis globally and in Iran. Rainfall was positively associated with VL in northeastern [35] and southwestern [16] Iran but negatively associated with VL in northwestern [28] and central [36] Iran and with CL in southwestern [10] and western [15] Iran. However, this was not recognized as an associated factor in a study in southern Iran [26]. Shirzadi et al. [37] investigated the impact of climatic factors on CL in a large area of northeastern/eastern Iran including North Khorasan, Razavi Khorasan (northeast), and South Khorasan (east). In this region, rainfall decreases from the mountainous regions in North Khorasan to the deserts of South Khorasan, with CL rates ~ 4.5 times as high in the northeast as in the east [37]. This lack of rainfall dependence is probably due to the reliance on underground water to support life in this arid region, where sand flies likely exist on the limited humidity provided by water pulled from underground sources. Similarly, in a semi-arid region in southern Iran, rainfall was not shown as a factor associated with CL spread [26].

## Conclusions

We have shown that urban setting, agricultural land cover, and the number of days with high-velocity wind were the most important determinants of the distribution of CL in the desert regions of South Khorasan in eastern Iran. Close human vector contact is promoted by a higher population density in urban areas and farming in agricultural land. Wind may spread the vectors and also allows them to concentrate and seek shelter in buildings to establish new niches. Low elevation probably sustains sand fly survival because of the underground water sources. *Leishmania tropica* is the main species in South Khorasan, and control strategies centered on reducing human vector contact should be focused on the at-risk zones.

## Data Availability

All data generated or analyzed during this study are included in this published article.

## Abbreviations

CL: Cutaneous leishmaniasis
MAR: Mean annual rainfall
MAH: Mean annual humidity
MAT: Mean annual temperature
MaxMAT: Maximum annual temperature
MinMAT: Minimum annual temperature
MAWD: Mean annual high-velocity wind days
MAFD: Mean annual frosty days
MASD: Mean annual snowy days
ZCL: Zoonotic cutaneous leishmaniasis
GIS: Geospatial information systems
RS: Remote sensing
PCR: Polymerase chain reaction
IDW: Inverse distance weighted
DEM: Digital elevation model
VL: Visceral leishmaniasis
